# PRESIDENTIAL SYMPOSIUM: EXTENDING HEALTHSPAN AND LONGEVITY

**DOI:** 10.1093/geroni/igad104.0455

**Published:** 2023-12-21

**Authors:** Blanka Rogina, Rafael de Cabo

## Abstract

Aging is associated with a functional decline in physiological, metabolic, proliferative, and tissue homeostasis leading to deterioration at an organismal level and an increase in risk for disease and death. To prevent or postpone such deterioration, genetic, pharmacological, and nutritional interventions have been successfully utilized, which lead to preserved healthspan and extended longevity. This symposium will discuss new approaches to target aging and the mechanisms underlying interventions that extend healthspan and longevity. The speakers of this symposium include internationally distinguished aging researchers who have dedicated their research to reveal new approaches to extending healthy lifespan. Rafael de Cabo, from NIA, Co-Chair and speaker in this symposium, will talk about the effects of calorie restriction on physiology, metabolism and health in a variety of organisms. Ana Gomes, from Moffitt Cancer Center, will talk about how aging promotes cancer resistance to therapy. Vishwa Deep Dixit, from Yale University, will discuss immunometabolic checkpoints of aging and lessons learned from CALERIE studies in humans. Payel Sen, from NIA, will present her studies on epigenetic mechanisms underlying tissue aging.

